# Management of necrotizing myositis in a field hospital: a case report

**DOI:** 10.1186/1757-7241-17-20

**Published:** 2009-04-18

**Authors:** Ramanathan Saranga Bharathi, Vinay Sharma, Rohit Sood, Arunava Chakladar, Pragnya Singh, Deep Kumar Raman

**Affiliations:** 1 Department of Surgery, 60 Parachute Field Hospital, C/O 56 APO, 904060, India; 2 Department of Surgery, Military Hospital, Agra Cantonment, Uttar Pradesh, 282002, India; 3 Department of Anesthesia, Military Hospital, Agra Cantonment, Uttar Pradesh, 282002, India; 4 Department of Pathology, Military Hospital, Agra Cantonment, Uttar Pradesh, 282002, India

## Abstract

Necrotizing myositis is a rare and fatal disease of skeletal muscles caused by group A beta hemolytic streptococci (GABHS). Its early detection by advanced imaging forms the basis of current management strategy. Paucity of advanced imaging in field/rural hospitals necessitates adoption of management strategy excluding imaging as its basis. Such a protocol, based on our experience and literature, constitutes:

i. Prompt recognition of the clinical triad: disproportionate pain; precipitous course; and early loss of power- in a swollen limb with/without preceding trauma.

ii. Support of clinical suspicion by 2 ubiquitous laboratory tests: gram staining- of exudates from bullae/muscles to indicate GABHS infection; and CPK estimation- to indicate myonecrosis.

iii. Replacement of empirical antibiotics with high intravenous doses of sodium penicillin and clindamycin

iv. Exploratory fasciotomy: to confirm myonecrosis without suppuration- its hallmark

v. Emergent radical debridement

vi. Primary closure with viable flaps – unconventional, if need be.

## Introduction

Necrotizing myositis (NM) is a rare disease of skeletal muscles caused by group A beta hemolytic streptococcus (GABHS) [1]. Although, considered uniformly fatal few years ago [1,2], its early detection by emergent magnetic resonance imaging (MRI)/computerized tomography (CT) has proved pivotal in its successful treatment and hence forms the cornerstone of current management strategy [3,4]. However, paucity of advanced imaging in field/rural hospitals necessitates adoption of management protocol excluding imaging as its basis. We attempt to expound such a protocol based on our experience with successful management of two cases with extensive disease and literature.

## Case reports

### Case 1

**Figure 1:**
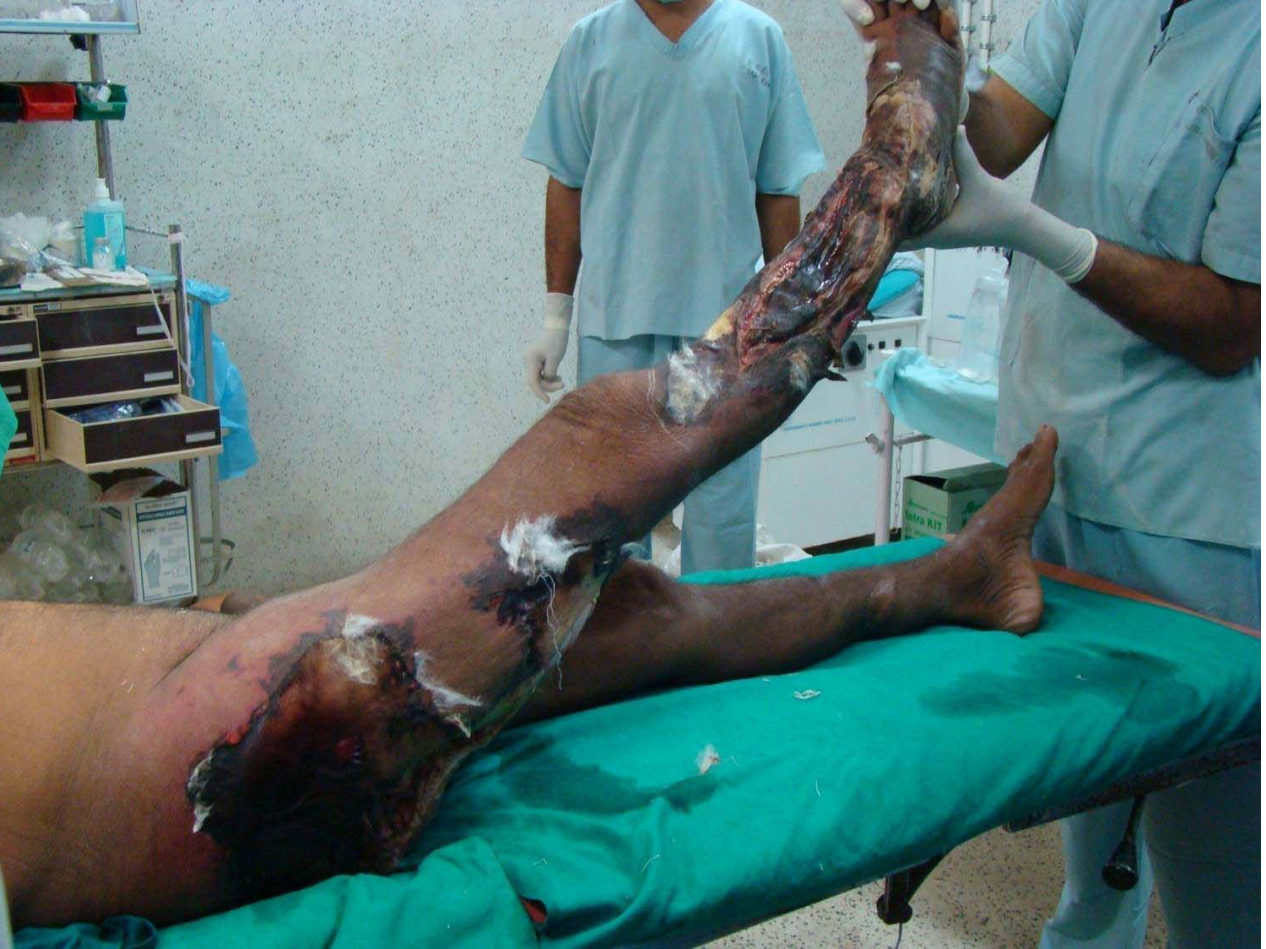
**Photograph showing the extent of involvement sparing the anterior compartment, with fasciotomies revealing the myonecrosis with conspicuously absent suppuration**.

**Figure 2:**
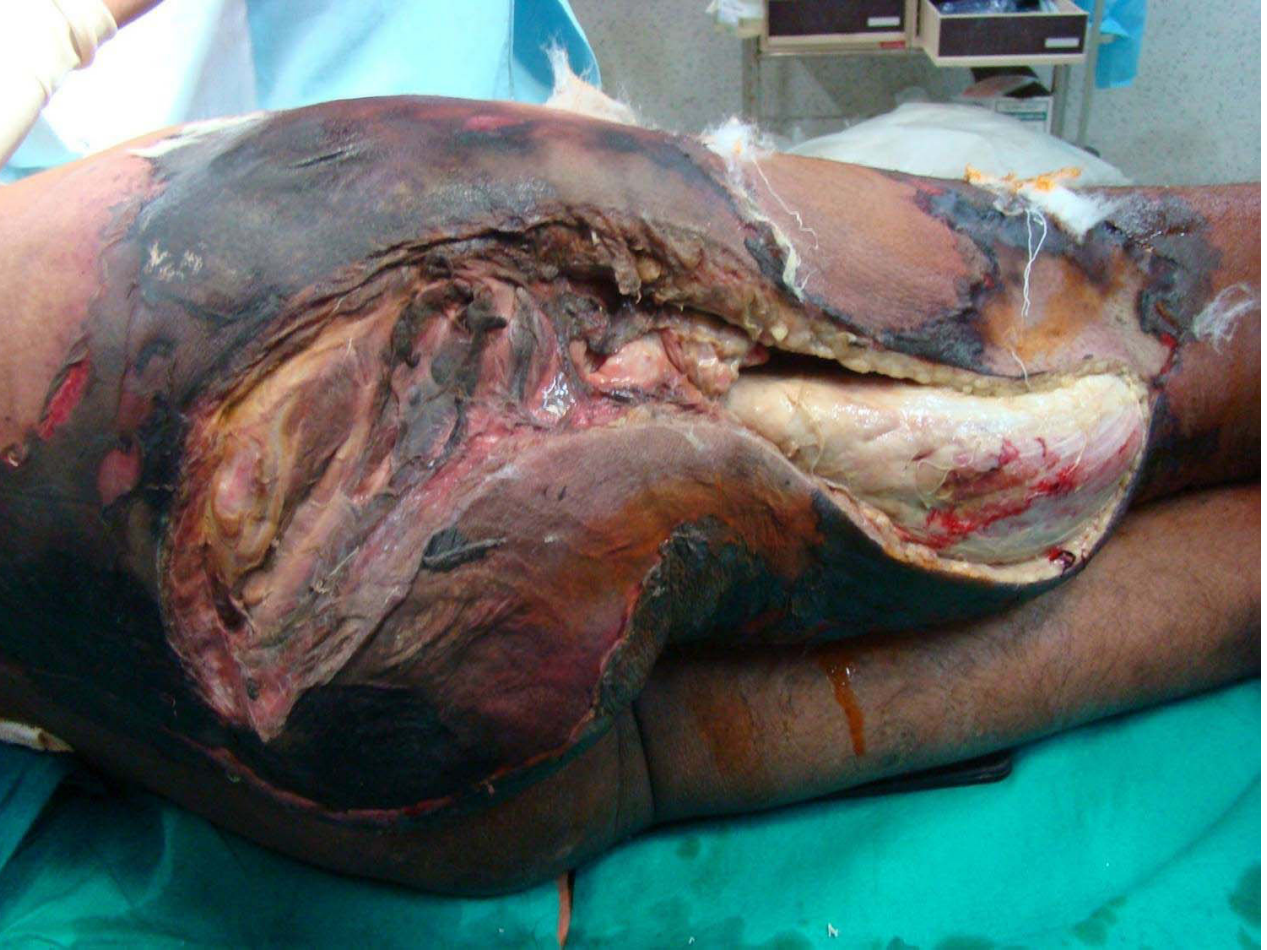
**Photograph showing the extent of involvement sparing the anterior compartment, with fasciotomies revealing the myonecrosis with conspicuously absent suppuration**.

**Figure 3:**
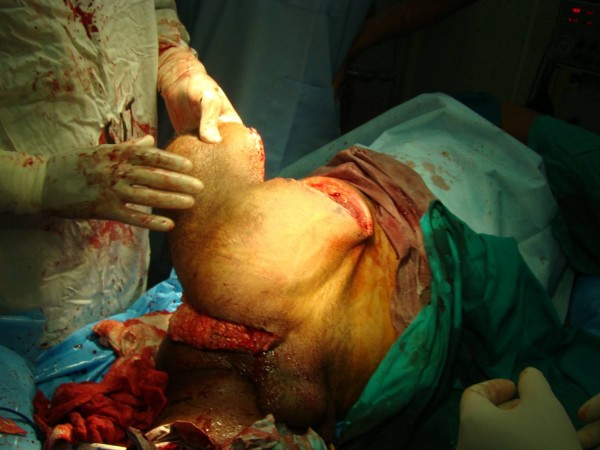
**Outer view of the harvested quadriceps flap**.

A previously healthy 56 years old male was brought with excruciatingly painful swelling of Rt lower limb, 2 days following trivial trauma to Rt foot. Patient had sepsis (tachycardia- 116/mt; hypotension- 88/52 mmHg, tachypnoea- 27/mt and low oxygen saturation- 84%) necessitating ventilatory and inotropic support (noradrenalin). Examination revealed swelling of entire Rt lower limb; few violaceous bullae; cutaneous necrosis of Rt leg, posterior thigh and gluteal region (Figs. 1 &2); and absent crepitations on palpation. The peripheral arterial pulsations till foot were discernable by hand held Doppler but muscular power was 0/V. Laboratory investigations except creatine phospho kinase (CPK- 23000 IU/L) and leucocytes (15600/cumm) were normal. Plain x-ray of the limb showed soft tissue swelling without gas. Gram staining of aspirate from bullae isolated streptococci in short chains. Urgent bed side fasciotomy (Figs. 1 &2) revealed extensive myonecrosis sparing the anterior compartment of thigh. Pus was conspicuously absent. With clinical diagnosis of NM, Sodium penicillin- 1 MU/4 hrly and Clindamycin 600 mg/6 hrly were commenced. Emergency hip disarticulation was performed including excision of entire gluteal compartment. Primary closure was achieved using quadriceps myocutaneous flap based on femoral artery (Fig. 3, 4 &5). The patient could be weaned off the ventilatory and inotropic support within 24 hours. GABHS cultured from the excised muscles were sensitive to penicillin, clindamycin and amikacin. The histopathology (Figs. 6 &7) revealed extensive coagulative necrosis; absent pus; dense infiltration of muscles and muscular arteries with leucocytes and GABHS confirming the diagnosis. Patient was discharged on complete recovery after 2 weeks.

**Figure 4:**
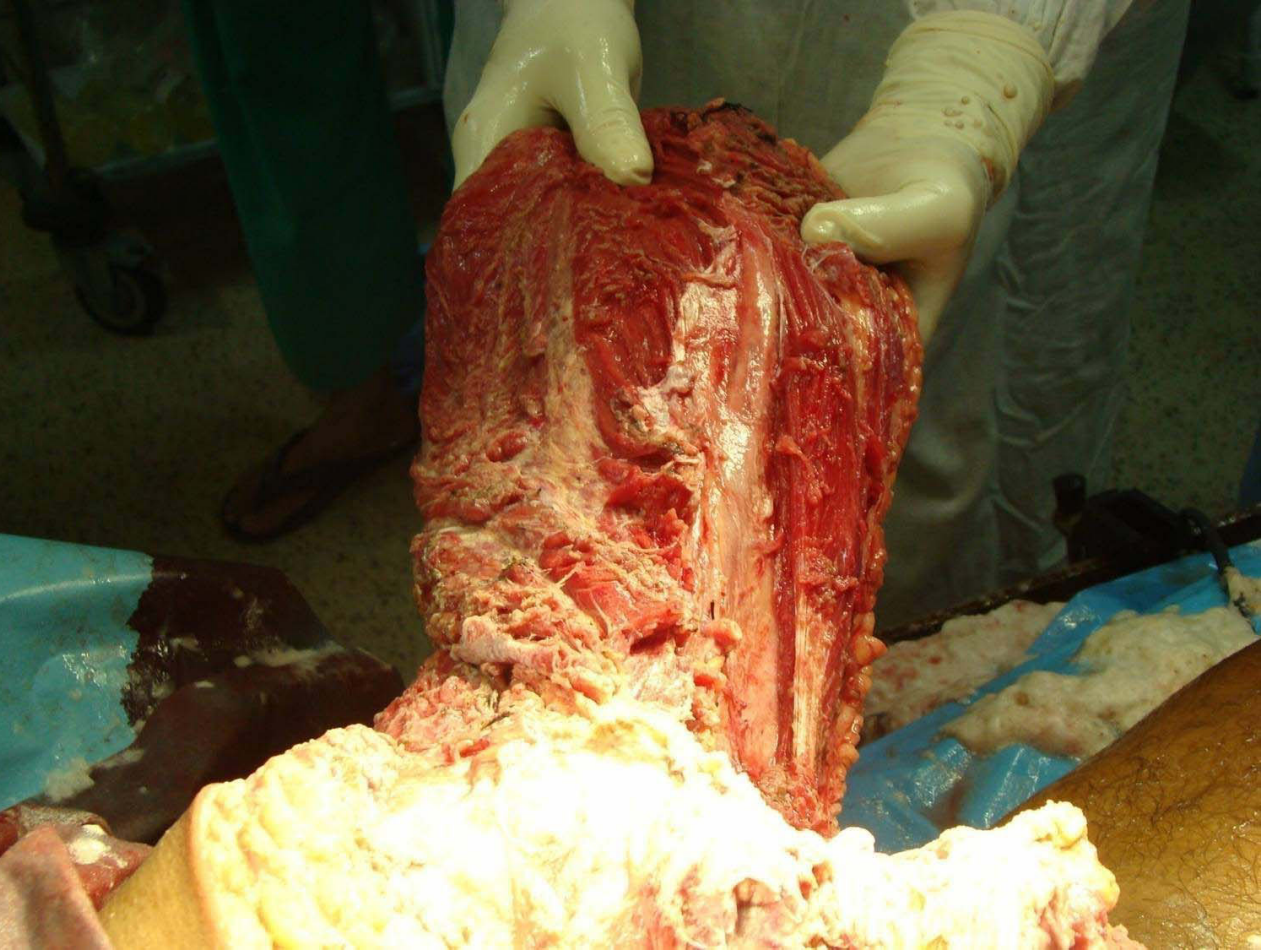
**Inner view of the quadriceps flap showing the femoral vessels**.

**Figure 5:**
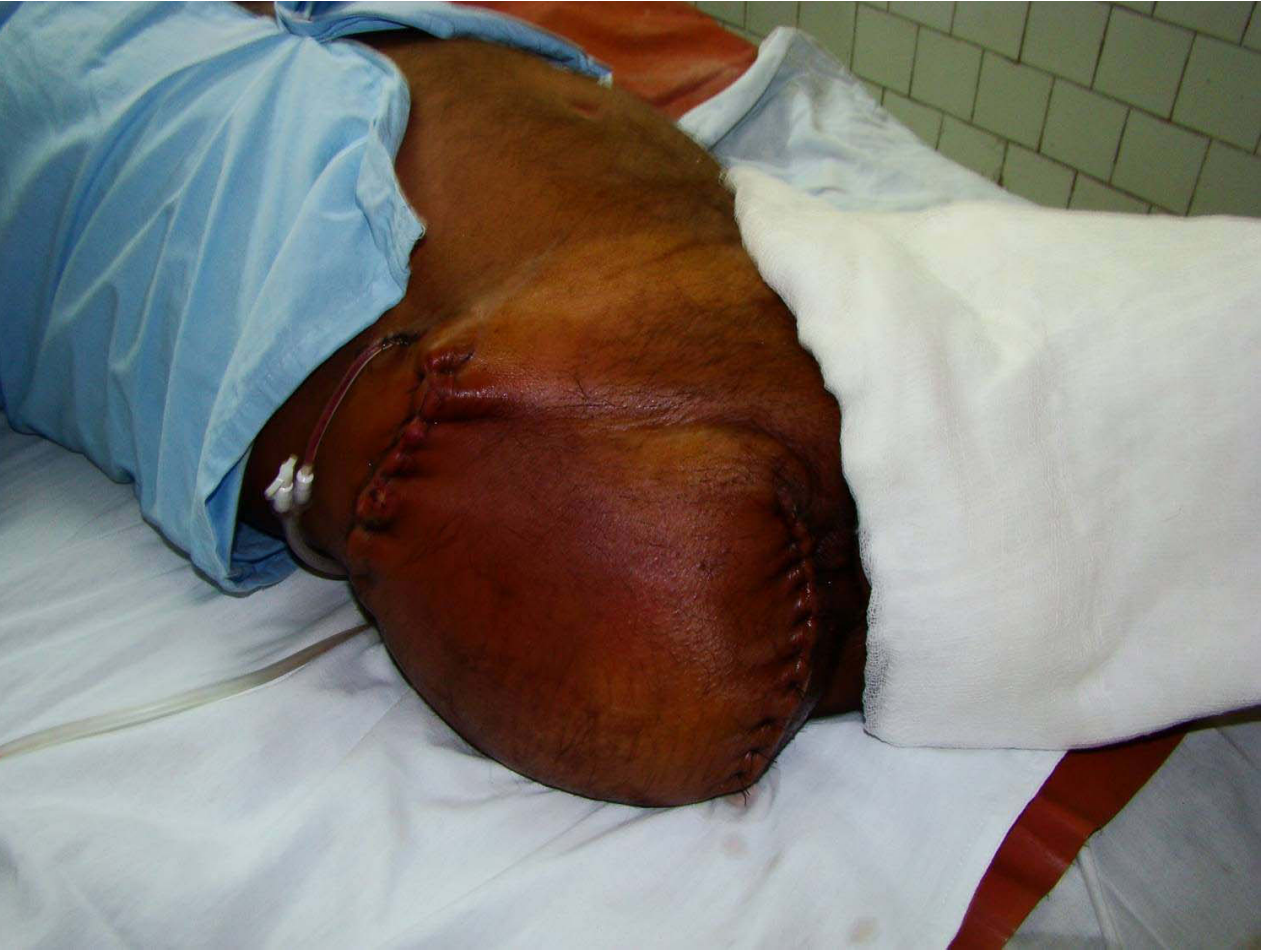
**Post operative photo showing the viable quadriceps flap**.

**Figure 6:**
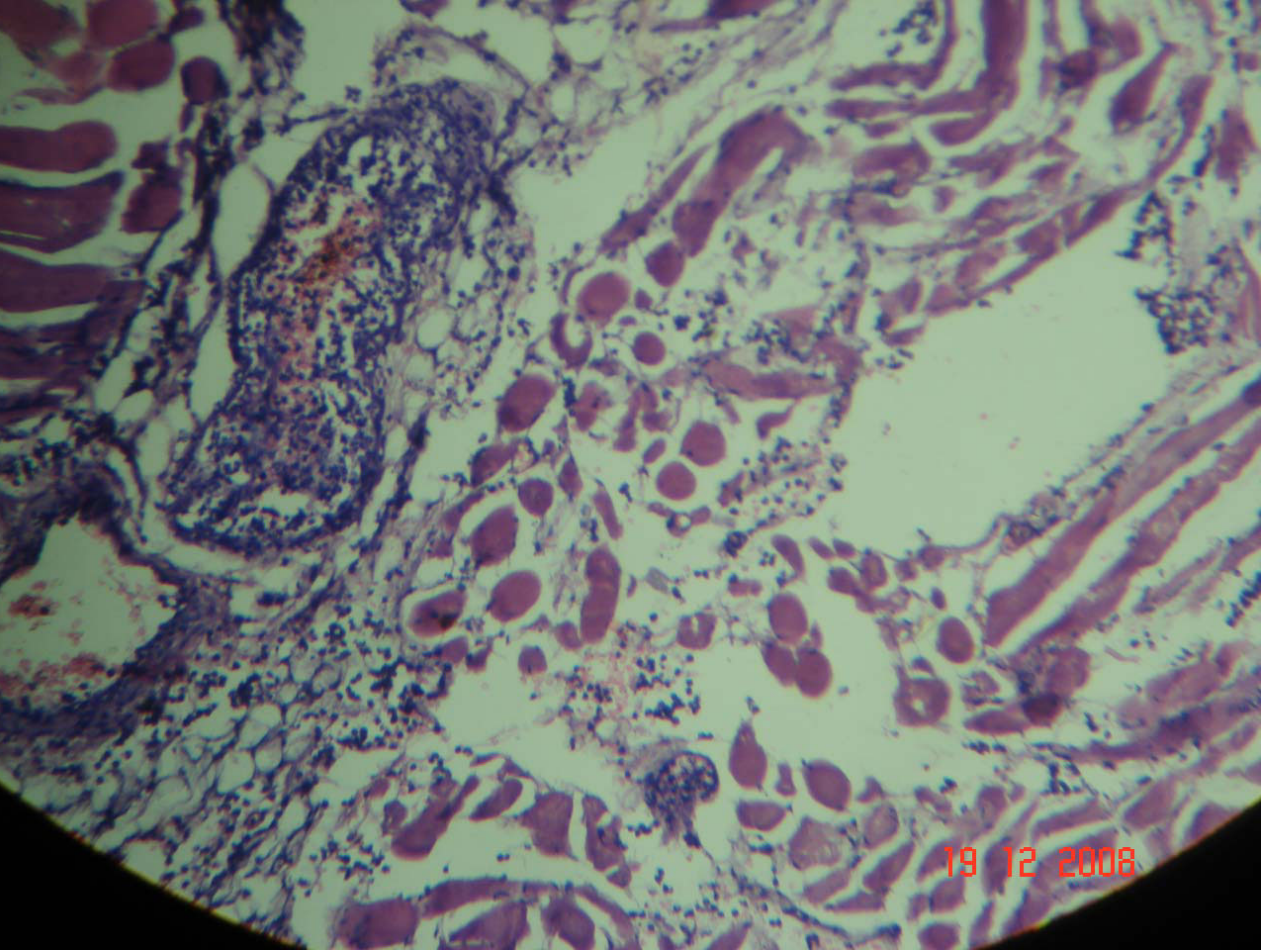
**Low power microscopic view depicting leucocytic infiltration of muscles and vessels**.

**Figure 7:**
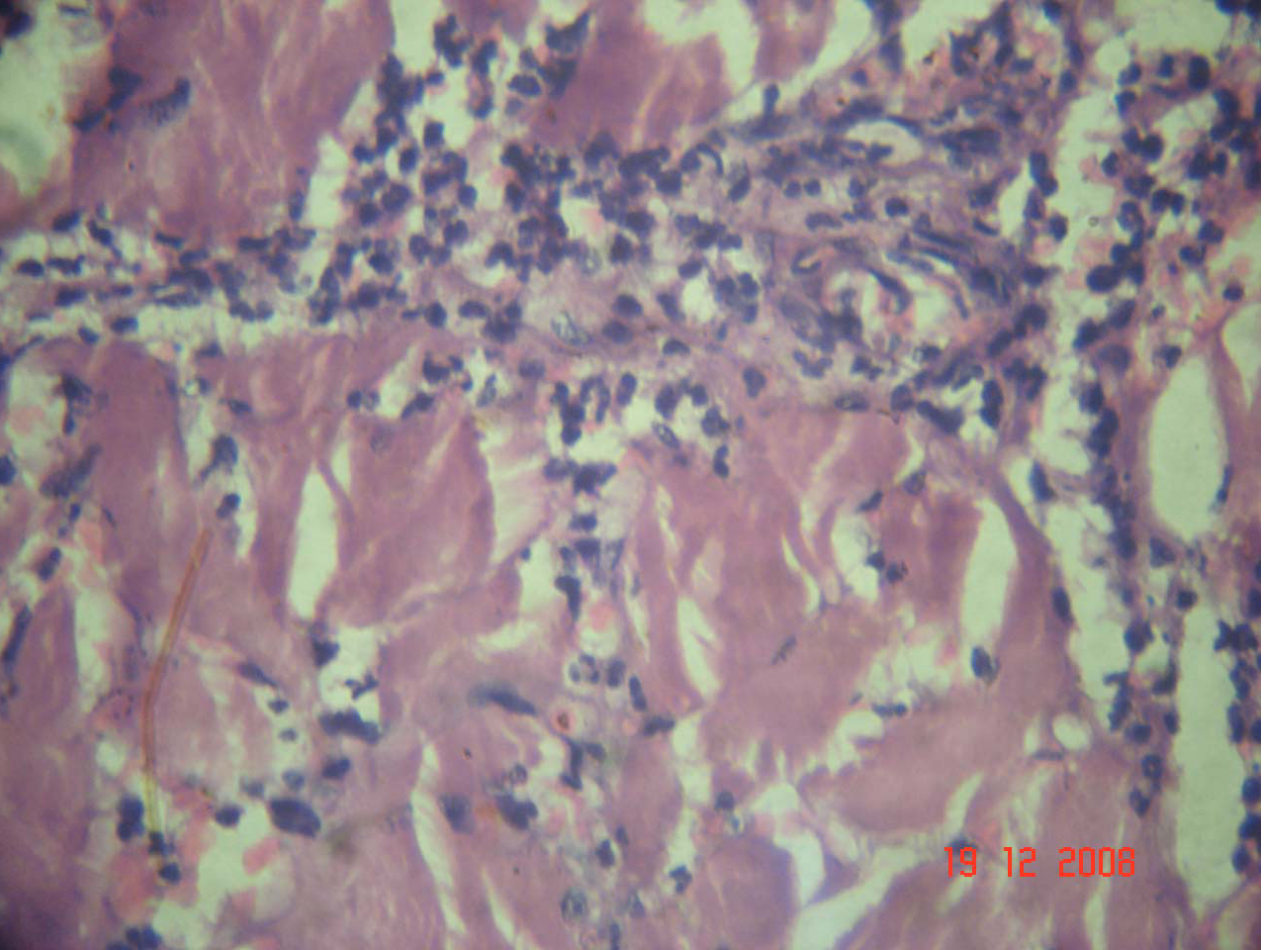
**High power microscopic view showing coagulative myonecrosis; absent pus; and dense leucocytic infiltration**.

### Case 2

**Figure 8:**
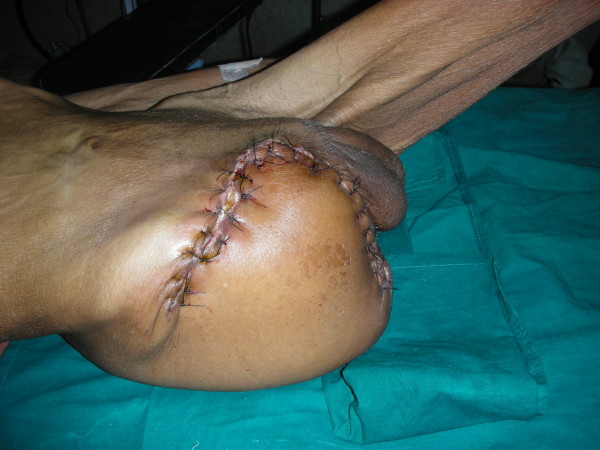
**Photograph showing viable lateral flap based on tensor fascia lata**.

A previously healthy 86 yrs old male was admitted as a case of cellulitis of Rt foot following 2 day old farm injury to 3^rd ^toe. He developed unbearable pain and swelling of the entire Rt lower extremity and within 24 hrs of admission. Examination revealed stable vital signs; barring few violaceous bullae the skin was entirely normal; peripheral arterial pulsations were palpable but muscular power was surprisingly lost; and crepitations were absent on palpation. Except CPK (18000 IU), laboratory investigations were normal. Plain x-ray of the limb showed soft tissue swelling without gas. Aspirate from the bullae isolated gram positive cocci in short chains. Sodium penicillin- 1 MU/4 hrly and Clindamycin 600 mg/6 hrly were commenced. Exploratory fasciotomy revealed myonecrosis of the entire lower limb sparing the gluteal compartment, with conspicuously absent pus. Emergency hip disarticulation was performed and primary closure achieved using tensor fascia lata based myocutaneous flap (Fig. 8). Histopathology confirmed NM. Excepting stitch abscess near anal opening, patient had remarkable recovery and could be discharged within 2 weeks.

## Disscussion

GABHS, a facultative anaerobe, causes myriad infections- from trivial cellulitis/lymphangitis to sinister toxic shock syndrome/endocarditis [5].

In addition, its propensity for causing necrotizing infections – necrotizing fascitis (NF), pyomyositis and NM, has earned it notoriety of '*flesh eating bacteria*' [1,5]. Among all, the least common (< 40 reported cases till date) [5] but most life threatening is NM, caused by the M1 and M3 subtypes of GABHS, which particularly are virulent by virtue of their antiphagocytic properties [4,5].

In addition to extensive local tissue destruction by releasing hyaluronidase, streptolysin and proteases, the pathogen causes spiraling systemic effects by amplifying pyrogenic exotoxins A and B which in turn activate the complement, histamine, kinin and lymphokine cascades leading to early multi organ dysfunction syndrome (MODS) [6,7]. Hence, survival in cases presenting late or with extensive disease and systemic manifestations has been uniformly disappointing (40%–100% mortality), despite robust treatment [5,7,8].

NM is characterized by rapid and extensive coagulative myonecrosis coupled with obliteration of muscular arteries with dense infiltration of leucocytes and GABHS (Figs. 6 &7) [1-5]. The distinctive feature differentiating this condition from other bacterial myositis is conspicuous *absence of pus *[1-5]. The skin and subcutaneous tissues are characteristically spared, initially, in contrast to the more common NF [1-5].

NSM affects previously healthy individuals irrespective of age [7]. Often, there is history of preceding trauma/infection, remote from site of affliction, which acts as the portal of entry, but is usually trivial and is recollected only in hindsight [1-5]. Though, predilection for proximal muscles of the lower limb has been observed [7], areas as diverse as tongue and arm/shoulder girdle have been involved [1,9].

There is scarcity of characteristic clinical features early in its course as complaints are common to varied conditions, such as, phlebothrombosis, hamstring pull, bursitis, cellulitis, lymphangitis and pyomyositis, rendering early diagnosis difficult [1-5,7,10]. This often results in fatal delay in initiation of appropriate management [7].

Barring few violaceous bullae the overlying skin is surprisingly normal, till late, and is shockingly disproportionate to the extent of underlying myonecrosis [7]. By the time skin necrosis is evident almost the entire extremity is irretrievably ruined (Fig. 1 and Fig. 2).

The only features that give out clues to early diagnosis are: early loss of muscular power (owing to early myonecrosis) unexplained by the other common conditions; precipitous course; and pain disproportionate to clinical signs (akin to mesenteric vascular infarction). A high index of suspicion is necessary to recognize this triad to diagnose this condition early. Though, acute limb ischemia and clostridial myonecrosis share all these features, but discernible peripheral pulsations and absent crepitations/air on plain x-rays help in their differentiation.

As the rapidity of infectious spread exceeds the body's ability to respond, the laboratory investigations are predominantly normal, initially, including the leucocytes count [1,5,7]. The only early marker which divulges underlying myonecrosis is raised CPK [1,4,5,7].

As the disease advances, multitude of abnormalities are detected, such as, myoglobinuria; raised polymormhonuclear leucocytosis; azotemia, etc. which are non specific and are more indicative of the onset of MODS than myositis *per se *[1,4,5,7].

Ubiquitous laboratory investigation significant enough to guide the management is- gram staining of fluid aspirated from bullae/muscles [7]. Isolation of streptococci (signifying GABHS infection) coupled with raised CPK (signifying myonecrosis) is, in our opinion, indicator enough for adoption of aggressive surgical management and change to high doses of specific antibiotics- combination of sodium penicillin and clindamycin [4], from empirically commenced ones. Culture and antibiotic sensitivity of the aspirates would, no doubt, be more specific/confirmatory but entails delaying specific treatment for 24–48 hours which might prove fatal.

CT/MRI, if available and done in time, can not only diagnose the condition early by revealing its singular hallmark – *myonecrosis without suppuration*, but also aid in differentiating the condition from confounding ones such as pyomyositis, clostridial myonecrosis, acute limb ischemia and phlegmasia cerulea dolens [3-5,7]. They also provide the road map for precise debridement by exclusively delineating the involved muscles [3,4]. Therefore, advanced imaging justly forms the basis of the current management strategy [3,4]. However, obtaining emergent CT/MRI is not feasible in rural settings and shifting the patient for the same entails loss of precious time as the entire spectrum of its course, from onset to development of MODS, telescopes into 2–3 days, at best [7].

Immediate '*exploratory fasciotomy*' (Fig 1&2), in our experience, renders the diagnosis splendidly clear, without the need for CT/MRI, by revealing myonecrosis with characteristic lack of pus. Additional incisions on the muscles confirm the absence of perfusion due to obliteration of muscular arteries by leucocytic infiltration. This simple procedure not only clinches the diagnosis, but also relieves the compartment pressures, decelerating the rapidity and extent of necrosis, providing the much needed time for resuscitation and planning the management. Additionally, the fluid that oozes from the muscles provides an additional uncontaminated sample for gram stain/culture [4,8].

Moreover, this procedure would prove therapeutic should the diagnosis turns out to be pyomyositis – by aiding drainage of pus; or necrotizing fascitis- by guiding the plane of debridement [3].

On establishing the diagnosis it is imperative to debride both emergently and radically [1,4] lest one might court failure due to the left over infected tissues which are well capable of further extension and triggering the cascades outlined earlier [1,7].

Some papers reporting successful salvage describe leaving the wound open and debriding conservatively followed by repeated debridements, when faced with further extension [1,4,9]. Such attempts, though well intentioned to save limb, are doomed to fail in the field/rural settings due reasons outlined earlier specifically due to paucity of advanced imaging for reassessment and critical care.

It is preferable to achieve primary closure as nosocomial cross infection between patients is but a rule in the wounds left open in the rural settings of the developing world. Unlike elective surgeries where appropriate flap cover can be planned, pattern of necrosis in NSM is unpredictable and the surgery emergent. Hence, classical flaps, such as, long posterior flap/fish mouth flaps for hip disarticulation may not be possible and one may have to resort to the use of unconventional flaps based on availability of viable tissues.

Involvement of all but the anterior compartment permitted use of long anterior quadriceps flap based on femoral artery (Fig. 3, 4, 5) in the first case. Though, an uncommon flap, predominantly employed for covering defects created by hemi-pelviectomy for sacral/gluteal tumors, it is a sturdy flap with excellent vascularity and is bulky enough to provide cushion for the exposed bones of the pelvis [11].

In the second case the involvement of all but the gluteal compartment rendered possible only a viable lateral flap based on tensor fascia lata (Fig. 8). Sparing the lateral circumflex iliac branch of femoral artery, while ligating the femoral vessels, is imperative for ensuring viability of the flap [12]. Basic knowledge of reconstructive surgery is helpful in successful salvage.

Critical care, intravenous immunoglobulin and hyperbaric therapy are definitely desirable [5-7], when indicated, but may be unnecessary if aggressive treatment protocol outlined above is adopted.

## Conclusion

Advanced cases of NM can be salvaged in field/rural hospitals, even in the absence of advanced imaging by adopting the outlined protocol: i. Recognition of the clinical triad – disproportionate pain; precipitous course; and loss of power- in a swollen limb with/without preceding trauma. ii. Detection of GABHS in gram staining of aspirates coupled with raised serum CPK. iii. Focused high intravenous doses of penicillin and clindamycin. iv. Exploratory fasciotomy with incision of muscles to confirm myo-necrosis without suppuration. v. Emergent radical debridement. vi. Primary closure using available tissues/flaps- unconventional, if need be.

## Consent

Written informed consent was obtained from the patient for publication of this case report and accompanying images. A copy of the written consent is available for review by the Editor-in-Chief of this journal.

## Competing interests

The authors declare that they have no competing interests.

## Authors' contributions

RSB, VS, RS and AC were members of the surgical team who operated upon the patients. RSB conceptualized this paper, carried out the review of literature and drafted the manuscript. PS and DKR were the pathologists/microbiologists who contributed to the laboratory studies as well as to the manuscript. RSB, VS and RS did the final editing before submission.

## References

[B1] Hird B, Byrne K (1994). Gangrenous streptococcal myositis: case report. J Trauma.

[B2] Subramanian KN, Lam KS (2003). Malignant necrotizing streptococcal myositis: a rare and fatal condition. J Bone Joint Surg (Br).

[B3] Tang WM, Wong JWK, Wong LLS, Leong JCY (2001). Streptococcal necrotizing myositis: the role of magnetic resonance imaging. J Bone Joint Surg (Am).

[B4] Dalal M, Sterne G, Murray DS (2002). Streptococcal myositis: a lesson. Br J Plast surg.

[B5] Saranga Bharathi R, Kalmath M, Singh KJ, Mohan PVR, Chaudhry R (2009). Streptococcal glossal myonecrosis – Is conservative treatment possible?. J Oral Maxillofac Surg.

[B6] Cunningham MW (2000). Pathogenesis of group A streptococcal infections. Clin Microbiol Rev.

[B7] Saranga Bharathi R, Agarwal A, Singh KJ, Gambhir RPS, Mohan PVR, Chaudhry R (2009). Necrotizing streptococcal myositis. ANZ J Surg.

[B8] Marck KW, den Hollander H, Grond AJ, Veenendaal D (1996). Survival after necrotizing streptococcal myositis: a matter of hours. Eur J Surg.

[B9] Doebelling BN, Wenzel RP (1989). Spontaneous streptococcal gangrenous myositis. South Med J.

[B10] Kang N, Antonopoulos D, Khanna A (1998). A case of streptococcal myositis (misdiagnosed as hamstring injury). J Accid Emerg Med.

[B11] Larson DL, Liang MD (1983). The quadriceps musculocutaneous flap: a reliable, sensate flap for the hemipelvectomy defect. Plast Reconstr Surg.

[B12] McGregor IA, McGregor AD (1995). Fundamental techniques of plastic surgery.

